# Supplementary figures and images for: Proteomic profiling of serum extracellular vesicles identifies diagnostic markers for echinococcosis
Source: PLoS Negl Trop Dis. 2022 Oct 7;16(10):e0010814. doi: 10.1371/journal.pntd.0010814 (PMC9581430; doi:10.1371/journal.pntd.0010814)

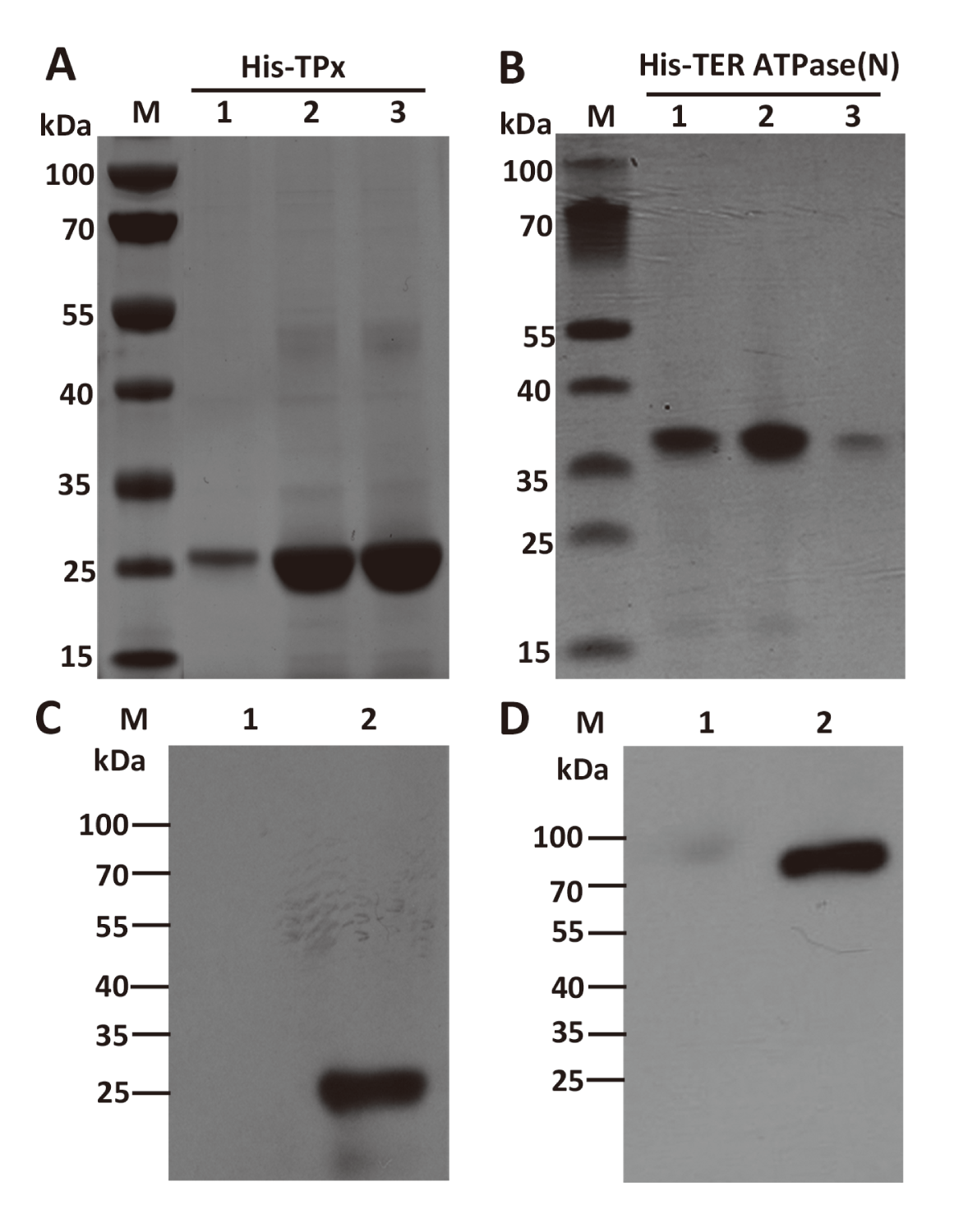

Supplement: S1 Fig — (A) and (B) SDS-PAGE analysis of purified recombinant TPx-1 or TER ATPase (truncated).(C) Western blotting analysis of native TPx-1 using anti-TPx-1 polyclonal antibodies. (D) Western blotting analysis of native TER ATPase using anti-TER ATPase polyclonal antibodies. (TIF) [file pntd.0010814.s001.tif]
