# Supplementary material for: Proteomic profiling of serum extracellular vesicles identifies diagnostic markers for echinococcosis
Source: PLoS Negl Trop Dis. 2022 Oct 7;16(10):e0010814. doi: 10.1371/journal.pntd.0010814 (PMC9581430; doi:10.1371/journal.pntd.0010814)
Supplement: S1 Table — (DOCX) [file pntd.0010814.s002.docx]

| Gene | Forward primer (5’ - 3’) | Reverse primer (5’ - 3’) |
| --- | --- | --- |
| VCP | GAGCTCATGTCTGCCTCGTCAGGAAATC | GTCGACCTCAACCGATGTCGTCGTAGCCAAC |
| VCP-N | GAGCTCATGTCTGCCTCGTCAGGAAATC | GTCGACCTCACGAATACAAGTCCTCATCC |
| TPx-1 | GGATCCATGGTTGCTCTTGTTGGG | GTCGACCTCACGAGCTCATGAACGAC |

S1 Table Primers used in the study
