# Supplementary material for: Proteomic profiling of serum extracellular vesicles identifies diagnostic markers for echinococcosis
Source: PLoS Negl Trop Dis. 2022 Oct 7;16(10):e0010814. doi: 10.1371/journal.pntd.0010814 (PMC9581430; doi:10.1371/journal.pntd.0010814)
Supplement: S2 Table — (DOCX) [file pntd.0010814.s003.docx]

| **S2 Table Summary of Proteins identified of serum-derived exosomes** | | | | | |
| --- | --- | --- | --- | --- | --- |
| **Protein_ID** | **Molecular Weigh (kD)** | **Num.of unique peptide** | **Num.of spectrum** | **Description** | **iBAQ** |
| P07724\|ALBU | 68.65 | 16 | 82 | Serum albumin | 135651 |
| Q921I1\|TRFE | 76.67 | 10 | 29 | Serotransferrin | 458805.8 |
| Q91X72\|HEMO | 51.29 | 5 | 26 | Hemopexin | 374742.6 |
| P11276\|FINC | 272.37 | 15 | 17 | Fibronectin | 50564.4 |
| Q922U2\|K2C5 | 61.73 | 11 | 17 | Keratin, type II cytoskeletal 5 | 204144.9 |
| P01872\|IGHM | 49.94 | 7 | 17 | Immunoglobulin heavy constant mu | 523805.5 |
| Q9QUM0\|ITA2B | 112.61 | 8 | 17 | Integrin alpha-IIb | 189033.8 |
| P02535\|K1C10 | 57.74 | 5 | 16 | Keratin, type I cytoskeletal 10 | 1369448 |
| Q9QWK4\|CD5L | 38.84 | 7 | 16 | CD5 antigen-like | 458757.3 |
| P08226\|APOE | 35.84 | 9 | 15 | Apolipoprotein E | 656141.8 |
| E9Q557\|DESP | 332.71 | 14 | 14 | Desmoplakin | 13545.07 |
| P01027\|CO3 | 186.37 | 5 | 12 | Complement C3 | 40064.76 |
| P02088\|HBB1 | 15.83 | 3 | 11 | Hemoglobin subunit beta-1 | 167921.7 |
| P07759\|SPA3K | 46.85 | 3 | 11 | Serine protease inhibitor A3K | 112754.8 |
| Q91ZX7\|LRP1 | 504.41 | 11 | 11 | Prolow-density lipoprotein receptor-related protein 1 | 7598.883 |
| Q60841\|RELN | 387.25 | 10 | 11 | Reelin | 16844.09 |
| Q00623\|APOA1 | 30.60 | 5 | 11 | Apolipoprotein A-I | 1317365 |
| A2AVA0\|SVEP1 | 387.20 | 9 | 10 | Sushi, von Willebrand factor type A, EGF and pentraxin dom | 19027.13 |
| P03987\|IGHG3 | 43.90 | 2 | 10 | Ig gamma-3 chain C region OS=Mus musculus OX=10090 P | 113003.5 |
| Q02257\|PLAK | 81.75 | 9 | 10 | Junction plakoglobin | 27379.07 |
| P01029\|CO4B | 192.79 | 8 | 10 | Complement C4-B | 40564.33 |
| Q80YX1\|TENA | 231.66 | 9 | 9 | Tenascin | 28540.6 |
| P06909\|CFAH | 139.05 | 5 | 9 | Complement factor H | 23441.44 |
| Q9Z2K1\|K1C16 | 51.57 | 5 | 8 | Keratin, type I cytoskeletal 16 | 168204.1 |
| Q8BTM8\|FLNA | 281.05 | 6 | 8 | Filamin-A | 15769.42 |
| P26039\|TLN1 | 269.65 | 5 | 8 | Talin-1 | 4016.101 |
| P54116\|STOM | 31.36 | 2 | 8 | Erythrocyte band 7 integral membrane protein | 335111.2 |
| Q61838\|PZP | 165.75 | 7 | 8 | Pregnancy zone protein | 73902.21 |
| Q06890\|CLUS | 51.62 | 4 | 7 | Clusterin | 151489.8 |
| tr\|A0A068YB19\|A0 | 49.26 | 4 | 7 | Actin | 210737.5 |
| P04104\|K2C1 | 65.57 | 3 | 7 | Keratin, type II cytoskeletal 1 | 376301.9 |
| P20918\|PLMN | 90.75 | 4 | 7 | Plasminogen | 52630.96 |
| P41317\|MBL2 | 25.94 | 2 | 6 | Mannose-binding protein C | 113023 |
| P04919\|B3AT | 103.07 | 5 | 6 | Band 3 anion transport protein | 245742.3 |
| Q3TTY5\|K22E | 70.88 | 5 | 6 | Keratin, type II cytoskeletal 2 epidermal | 541689.4 |
| Q61147\|CERU | 121.07 | 4 | 6 | Ceruloplasmin | 139520.4 |
| Q9DBB9\|CPN2 | 60.44 | 4 | 5 | Carboxypeptidase N subunit 2 | 49645.22 |
| O08538\|ANGP1 | 57.48 | 5 | 5 | Angiopoietin-1 OS=Mus musculus | 52762.74 |
| Q9QWL7\|K1C17 | 48.13 | 3 | 5 | Keratin, type I cytoskeletal 17 | 58117.74 |
| Q9DBD0\|ICA | 76.72 | 4 | 5 | Inhibitor of carbonic anhydrase | 10197.08 |
| Q8VDD5\|MYH9 | 226.23 | 5 | 5 | Myosin-9 | 7851.345 |
| P98064\|MASP1 | 79.92 | 3 | 5 | Mannan-binding lectin serine protease 1 | 15518.83 |
| P23953\|EST1C | 61.02 | 3 | 5 | Carboxylesterase 1C | 49190.15 |
| O35598\|ADA10 | 83.91 | 5 | 5 | Disintegrin and metalloproteinase domain-containing protein | 25564.08 |
| P13020\|GELS | 85.89 | 4 | 5 | Gelsolin | 57770.39 |
| Q00896\|A1AT3 | 45.79 | 2 | 5 | Alpha-1-antitrypsin 1-3 | 103632.8 |
| P14106\|C1QB | 26.70 | 2 | 5 | Complement C1q subcomponent subunit B | 407578.4 |
| Q01279\|EGFR | 134.77 | 5 | 5 | Epidermal growth factor receptor | 14555.88 |
| Q64727\|VINC | 116.64 | 5 | 5 | Vinculin | 23782.11 |
| P19221\|THRB | 70.22 | 5 | 5 | Prothrombin | 33034.91 |
| O55222\|ILK | 51.34 | 3 | 5 | Integrin-linked protein kinase | 10486.02 |
| Q923D2\|BLVRB | 22.18 | 2 | 4 | Flavin reductase (NADPH) | 126393.5 |
| Q07076\|ANXA7 | 49.89 | 3 | 4 | Annexin A7 | 0 |
| P01898\|HA10 | 37.23 | 4 | 4 | H-2 class I histocompatibility antigen, Q10 alpha chain | 70133.88 |
| P17156\|HSP72 | 69.60 | 3 | 4 | Heat shock-related 70 kDa protein 2 | 35280.37 |
| Q8VDN2\|AT1A1 | 112.91 | 3 | 4 | Sodium/potassium-transporting ATPase subunit alpha-1 | 14815.06 |
| O88783\|FA5 | 247.08 | 4 | 4 | Coagulation factor V | 6184.192 |
| P04186\|CFAB | 84.95 | 3 | 4 | Complement factor B | 58505.39 |
| P29391\|FRIL1 | 20.79 | 4 | 4 | Ferritin light chain 1 | 42047.86 |
| Q91WP0\|MASP2 | 75.47 | 2 | 4 | Mannan-binding lectin serine protease 2 | 96448.51 |
| Q8CG16\|C1RA | 80.02 | 3 | 4 | Complement C1r-A subcomponent | 35361.91 |
| Q00724\|RET4 | 23.19 | 2 | 4 | Retinol-binding protein 4 | 35626.4 |
| P01592\|IGJ | 18.00 | 1 | 4 | Immunoglobulin J chai | 159371.4 |
| Q61730\|IL1AP | 65.70 | 3 | 4 | Interleukin-1 receptor accessory protein | 20005.81 |
| P50446\|K2C6A | 59.30 | 4 | 4 | Keratin, type II cytoskeletal 6A | 100800.9 |

| P01808\|HVM38 | 13.16 | 3 | 4 | Ig heavy chain V region T601 | 476006.6 |
| --- | --- | --- | --- | --- | --- |
| P35441\|TSP1 | 129.56 | 3 | 4 | Thrombospondin-1 | 29816.69 |
| P06728\|APOA4 | 45.00 | 2 | 3 | Apolipoprotein A-IV | 31538.02 |
| A6X935\|ITIH4 | 104.59 | 1 | 3 | Inter alpha-trypsin inhibitor, heavy chain 4 | 27682.2 |
| Q6IFX2\|K1C42 | 50.10 | 2 | 3 | Keratin, type I cytoskeletal 42 | 69787.02 |
| P29788\|VTNC | 54.81 | 2 | 3 | Vitronectin | 142071.7 |
| P09528\|FRIH | 21.05 | 1 | 3 | Ferritin heavy chain | 8838.502 |
| O54890\|ITB3 | 86.68 | 2 | 3 | Integrin beta-3 | 22830.58 |
| Q07797\|LG3BP | 64.45 | 2 | 3 | Galectin-3-binding protein | 4133.096 |
| Q64455\|PTPRJ | 136.68 | 3 | 3 | Receptor-type tyrosine-protein phosphatase eta | 26716.55 |
| Q8CG14\|CS1A | 76.81 | 2 | 3 | Complement C1s-A subcomponent | 16165.32 |
| P18527\|HVM56 | 10.65 | 2 | 3 | Ig heavy chain V region 914 | 42240.59 |
| Q61129\|CFAI | 67.22 | 1 | 3 | Complement factor I | 5938.161 |
| Q7TPR4\|ACTN1 | 103.00 | 2 | 3 | Alpha-actinin-1 | 3878.226 |
| A2AQ07\|TBB1 | 50.41 | 3 | 3 | Tubulin beta-1 chain OS | 32887.45 |
| P56480\|ATPB | 56.27 | 3 | 3 | ATP synthase subunit beta, mitochondrial | 14161.09 |
| P22599\|A1AT2 | 45.95 | 2 | 3 | Alpha-1-antitrypsin 1-2 | 351274.8 |
| P28665\|MUG1 | 165.19 | 2 | 3 | Murinoglobulin-1 OS | 64834.64 |
| P11680\|PROP | 50.29 | 2 | 3 | Properdin OS=Mus | 6885.115 |
| Q02105\|C1QC | 25.97 | 1 | 3 | Complement C1q subcomponent subunit C | 165045.8 |
| Q6IFZ6\|K2C1B | 61.32 | 1 | 3 | Keratin, type II cytoskeletal 1b | 266872.3 |
| Q3UV17\|K22O | 62.81 | 2 | 3 | Keratin, type II cytoskeletal 2 oral | 1130224 |
| P01630\|KV2A6 | 12.49 | 2 | 3 | Ig kappa chain V-II region 7S34.1 | 448510.2 |
| O70362\|PHLD | 93.20 | 3 | 3 | Phosphatidylinositol-glycan-specific phospholipase D | 8079.652 |
| Q8K1B8\|URP2 | 75.59 | 3 | 3 | Fermitin family homolog 3 | 87495.17 |
| E9Q414\|APOB | 509.11 | 2 | 3 | Apolipoprotein B-100 | 0 |
| O88844\|IDHC | 46.64 | 1 | 2 | Isocitrate dehydrogenase [NADP] cytoplasmic | 2925.378 |
| Q9EQK5\|MVP | 95.87 | 2 | 2 | Major vault protein | 9860.071 |
| P32261\|ANT3 | 51.97 | 1 | 2 | Antithrombin-III | 0 |
| P05366\|SAA1 | 13.76 | 2 | 2 | Serum amyloid A-1 protein | 8561.138 |
| P05064\|ALDOA | 39.33 | 2 | 2 | Fructose-bisphosphate aldolase A O | 37450.67 |
| P00920\|CAH2 | 29.01 | 1 | 2 | Carbonic anhydrase 2 | 7710.233 |
| P01900\|HA12 | 41.08 | 2 | 2 | H-2 class I histocompatibility antigen, D-D alpha chain | 16074.88 |
| Q01339\|APOH | 38.59 | 1 | 2 | Beta-2-glycoprotein 1 | 650.9385 |
| P04202\|TGFB1 | 44.28 | 1 | 2 | Transforming growth factor beta-1 proprotein | 2085.451 |
| P06800\|PTPRC | 144.75 | 2 | 2 | Receptor-type tyrosine-protein phosphatase C | 1137.667 |
| P24270\|CATA | 59.76 | 2 | 2 | Catalase | 8120.538 |
| P97429\|ANXA4 | 35.89 | 2 | 2 | Annexin A4 | 2689.875 |
| Q60994\|ADIPO | 26.79 | 2 | 2 | Adiponectin | 64295.53 |
| P48193\|41 | 95.85 | 2 | 2 | Protein 4.1 | 67611.46 |
| Q8BT60\|CPNE3 | 59.55 | 2 | 2 | Copine-3 | 4237.565 |
| Q68FD5\|CLH1 | 191.43 | 2 | 2 | Clathrin heavy chain 1 | 3182.191 |
| Q99K41\|EMIL1 | 107.52 | 2 | 2 | EMILIN-1 | 897.9359 |
| Q6P9J9\|ANO6 | 106.19 | 2 | 2 | Anoctamin-6 | 300.3362 |
| Q8K182\|CO8A | 66.04 | 2 | 2 | Complement component C8 alpha chain | 0 |
| P01837\|IGKC | 11.93 | 1 | 2 | Immunoglobulin kappa constant | 138815 |
| Q9EQP2\|EHD4 | 61.44 | 2 | 2 | EH domain-containing protein 4 | 4807.881 |
| Q8K558\|TRML1 | 33.50 | 2 | 2 | Trem-like transcript 1 protein | 17228.77 |
| Q6P069\|SORCN | 21.61 | 1 | 2 | Sorcin | 6993.052 |
| P97384\|ANX11 | 54.04 | 2 | 2 | Annexin A11 | 10075.03 |
| P06684\|CO5 | 188.76 | 2 | 2 | Complement C5 | 320.7496 |
| Q07968\|F13B | 76.15 | 2 | 2 | Coagulation factor XIII B chain | 23672.55 |
| Q8R411\|MYCT1 | 21.07 | 2 | 2 | Myc target protein 1 | 52089.69 |
| Q61495\|DSG1A | 114.52 | 2 | 2 | Desmoglein-1-alpha | 9990.589 |
| P56400\|GP1BB | 21.75 | 2 | 2 | Platelet glycoprotein Ib beta chain | 244092.9 |
| P01790\|HVM21 | 13.64 | 2 | 2 | Ig heavy chain V region M511 | 1825514 |
| P01642\|KV5A9 | 12.61 | 1 | 2 | Ig kappa chain V-V region L7 (Fragment) | 88363.96 |
| P03953\|CFAD | 28.04 | 1 | 2 | Complement factor D | 54606.44 |
| Q9ESB3\|HRG | 59.13 | 2 | 2 | Histidine-rich glycoprotein O | 107266.3 |
| Q61508\|ECM1 | 62.79 | 2 | 2 | Extracellular matrix protein 1 | 4280.889 |
| Q61268\|APOC4 | 14.28 | 2 | 2 | Apolipoprotein C-IV | 95911.01 |
| P11859\|ANGT | 51.96 | 2 | 2 | Angiotensinogen | 4162.528 |
| Q9JJN5\|CBPN | 51.81 | 2 | 2 | Carboxypeptidase N catalytic chain | 13612.19 |
| Q8K1I3\|SPP24 | 23.12 | 2 | 2 | Secreted phosphoprotein 24 | 0 |
| Q80Z71\|TENN | 172.98 | 2 | 2 | Tenascin-N | 166.4177 |
| P04945\|KV6AB | 11.71 | 1 | 2 | Ig kappa chain V-VI region NQ2-6.1 | 30792.45 |
| Q02357\|ANK1 | 204.10 | 2 | 2 | Ankyrin-1 | 644.8688 |
| Q03265\|ATPA | 59.72 | 2 | 2 | ATP synthase subunit alpha, mitochondrial | 8909.901 |
| O88947\|FA10 | 53.98 | 1 | 1 | Coagulation factor X | 2284.192 |
| P51863\|VA0D1 | 40.28 | 1 | 1 | V-type proton ATPase subunit d 1 | 560.185 |
| Q9QUM9\|PSA6 | 27.35 | 1 | 1 | Proteasome subunit alpha type-6 | 1210328 |

| P09055\|ITB1 | 88.17 | 1 | 1 | Integrin beta-1 | 11727.51 |
| --- | --- | --- | --- | --- | --- |
| O35955\|PSB10 | 29.04 | 1 | 1 | Proteasome subunit beta type-10 | 2927.864 |
| Q61247\|A2AP | 54.94 | 1 | 1 | Alpha-2-antiplasmin O | 18579.95 |
| Q5PR73\|DIRA2 | 22.48 | 1 | 1 | GTP-binding protein Di-Ras2 | 22515.18 |
| tr\|A0A068XXP8\|A0 | 26.30 | 1 | 1 | Adenylate kinase 2 mitochondrial | 3082.772 |
| Q08481\|PECA1 | 81.21 | 1 | 1 | Platelet endothelial cell adhesion molecule | 8755.556 |
| Q9CQW9\|IFM3 | 14.94 | 1 | 1 | Interferon-induced transmembrane protein 3 | 70367.44 |
| P18052\|PTPRA | 93.64 | 1 | 1 | Receptor-type tyrosine-protein phosphatase alpha | 2437.434 |
| Q99P58\|RB27B | 24.54 | 1 | 1 | Ras-related protein Rab-27B | 10728.28 |
| P39876\|TIMP3 | 24.17 | 1 | 1 | Metalloproteinase inhibitor 3 | 16569.47 |
| Q62009\|POSTN | 93.08 | 1 | 1 | Periostin | 734.3078 |
| P70290\|EM55 | 52.19 | 1 | 1 | 55 kDa erythrocyte membrane protein | 2816.775 |
| Q61781\|K1C14 | 52.83 | 1 | 1 | Keratin, type I cytoskeletal 14 | 0 |
| A2A6A1\|GPTC8 | 164.89 | 1 | 1 | G patch domain-containing protein 8 | 0 |
| Q8VCG4\|CO8G | 22.49 | 1 | 1 | Complement component C8 gamma chain | 29163.4 |
| P62259\|1433E | 29.16 | 1 | 1 | 14-3-3 protein epsilon | 11978.78 |
| Q64324\|STXB2 | 66.32 | 1 | 1 | Syntaxin-binding protein 2 | 0 |
| P06683\|CO9 | 61.96 | 1 | 1 | Complement component C9 | 0 |
| P29699\|FETUA | 37.30 | 1 | 1 | Alpha-2-HS-glycoprotein | 0 |
| Q9JL99\|CLC1B | 26.22 | 1 | 1 | C-type lectin domain family 1 member | 5128.537 |
| P06330\|HVM51 | 12.93 | 1 | 1 | Ig heavy chain V region AC38 205.12 | 0 |
| tr\|A0A068YJG0\|A0 | 137.33 | 1 | 1 | Expressed protein | 0 |
| P24063\|ITAL | 128.25 | 1 | 1 | Integrin alpha-L | 1553.375 |
| P49222\|EPB42 | 76.71 | 1 | 1 | Erythrocyte membrane protein band 4.2 | 9082.684 |
| Q6X893\|CTL1 | 73.02 | 1 | 1 | Choline transporter-like protein 1 | 78.59566 |
| P12246\|SAMP | 26.23 | 1 | 1 | Serum amyloid P-component | 183596.3 |
| P01869\|IGH1M | 43.36 | 1 | 1 | Ig gamma-1 chain C region, membrane-bound form | 5863.989 |
| Q924C1\|XPO5 | 136.88 | 1 | 1 | Exportin-5 | 0 |
| P32037\|GTR3 | 53.44 | 1 | 1 | Solute carrier family 2, facilitated glucose transporter membe | 39328.63 |
| tr\|A0A068YJ03\|A0 | 35.82 | 1 | 1 | Src y 3 domain | 23606.99 |
| P08103\|HCK | 59.09 | 1 | 1 | Tyrosine-protein kinase HCK | 3249.55 |
| E9PV24\|FIBA | 87.38 | 1 | 1 | Fibrinogen alpha chain | 2637.335 |
| P01680\|KV4A1 | 13.82 | 1 | 1 | Ig kappa chain V-IV region S107B | 3134737 |
| Q8BND5\|QSOX1 | 82.73 | 1 | 1 | Sulfhydryl oxidase 1 | 1277.694 |
| Q9R1P4\|PSA1 | 29.53 | 1 | 1 | Proteasome subunit alpha type-1 | 188.9861 |
| Q99JY9\|ARP3 | 47.33 | 1 | 1 | Actin-related protein 3 | 823.66 |
| tr\|A0A068XYC9\|A0 | 114.14 | 1 | 1 | Phd finger protein 10 OS=Echinococcus multilocularis OX= | 357.8385 |
| P18572\|BASI | 42.42 | 1 | 1 | Basigin | 60657.97 |
| P07356\|ANXA2 | 38.65 | 1 | 1 | Annexin A2 | 3075.149 |
| O70325\|GPX4 | 22.21 | 1 | 1 | Phospholipid hydroperoxide glutathione peroxidase | 6121.534 |
| P48036\|ANXA5 | 35.73 | 1 | 1 | Annexin A5 | 6295.104 |
| P98086\|C1QA | 25.96 | 1 | 1 | Complement C1q subcomponent subunit A | 11399.35 |
| P52480\|KPYM | 57.81 | 1 | 1 | Pyruvate kinase PKM | 124.4349 |
| Q61646\|HPT | 38.73 | 1 | 1 | Haptoglobin | 621611.6 |
| P03976\|KV2A5 | 12.38 | 1 | 1 | Ig kappa chain V-II region 17S29.1 | 614172.7 |
| O70439\|STX7 | 29.80 | 1 | 1 | Syntaxin-7 | 0 |
| Q8C166\|CPNE1 | 58.85 | 1 | 1 | Copine-1 | 7285.108 |
| Q8R146\|APEH | 81.53 | 1 | 1 | Acylamino-acid-releasing enzyme | 554.261 |
| O35173\|KCNS1 | 54.88 | 1 | 1 | Potassium voltage-gated channel subfamily S member 1 | 185.0831 |
| P61028\|RAB8B | 23.59 | 1 | 1 | Ras-related protein Rab-8B O | 606.3974 |
| P08228\|SODC | 15.93 | 1 | 1 | Superoxide dismutase [Cu-Zn] | 14822.56 |
| Q60790\|RASA3 | 95.93 | 1 | 1 | Ras GTPase-activating protein 3 | 3163.015 |
| Q02013\|AQP1 | 28.78 | 1 | 1 | Aquaporin-1 | 374527.1 |
| P58771\|TPM1 | 32.66 | 1 | 1 | Tropomyosin alpha-1 chain | 3484.926 |
| P21614\|VTDB | 53.56 | 1 | 1 | Vitamin D-binding protein | 28037.29 |
| Q9D6Z6\|IL36B | 20.86 | 1 | 1 | Interleukin-36 beta | 1685.858 |
| P0C0S6\|H2AZ | 13.54 | 1 | 1 | Histone H2A.Z | 0 |
| O70435\|PSA3 | 28.39 | 1 | 1 | Proteasome subunit alpha type-3 | 0 |
| Q9ET66\|PI16 | 53.62 | 1 | 1 | Peptidase inhibitor 16 | 2521.92 |
| O88634\|PAR4 | 42.76 | 1 | 1 | Proteinase-activated receptor 4 | 2219.848 |
| P21279\|GNAQ | 42.13 | 1 | 1 | Guanine nucleotide-binding protein G(q) subunit alpha | 2150.853 |
| P27773\|PDIA3 | 56.64 | 1 | 1 | Protein disulfide-isomerase A3 | 3278.629 |
| P08752\|GNAI2 | 40.46 | 1 | 1 | Guanine nucleotide-binding protein G(i) subunit alpha-2 | 0 |
| Q8R429\|AT2A1 | 109.35 | 1 | 1 | Sarcoplasmic/endoplasmic reticulum calcium ATPase 1 | 1075.668 |
| Q8CHB8\|TTLL5 | 147.62 | 1 | 1 | Tubulin polyglutamylase TTLL5 | 0 |
| P30987\|TA2R | 37.07 | 1 | 1 | Thromboxane A2 receptor | 144625.1 |
| Q80YC5\|FA12 | 65.66 | 1 | 1 | Coagulation factor XII | 0 |
| tr\|A0A068YD29\|A0 | 34.97 | 1 | 1 | PUR alpha protein | 0 |
| P01644\|KV5AB | 11.90 | 1 | 1 | Ig kappa chain V-V region HP R16.7 | 0 |
| P43252\|PI2R | 44.43 | 1 | 1 | Prostacyclin receptor | 973.8296 |
| Q8CFW1\|ANO2 | 114.06 | 1 | 1 | Anoctamin-2 | 4382.122 |

| P68368\|TBA4A | 49.89 | 1 | 1 | Tubulin alpha-4A chain | 9397.984 |
| --- | --- | --- | --- | --- | --- |
| O35930\|GP1BA | 80.01 | 1 | 1 | Platelet glycoprotein Ib alpha chain | 14670.69 |
| Q8R4V5\|OIT3 | 60.28 | 1 | 1 | Oncoprotein-induced transcript 3 protein | 0 |
| Q9CPV9\|P2Y12 | 39.45 | 1 | 1 | P2Y purinoceptor 12 | 86878.2 |
| tr\|A0A087VYG8\|A0 | 39.15 | 1 | 1 | Replication factor C subunit 4 | 26028.94 |
| P10518\|HEM2 | 36.00 | 1 | 1 | Delta-aminolevulinic acid dehydratase | 9325.663 |
| tr\|A0A068Y3Y9\|A0 | 392.71 | 1 | 1 | Hypothetical transcript | 0 |
| Q497I4\|KRT35 | 50.50 | 1 | 1 | Keratin, type I cuticular Ha5 | 637.4428 |
| P55065\|PLTP | 54.42 | 1 | 1 | Phospholipid transfer protein | 0 |
| O88200\|CLC11 | 36.43 | 1 | 1 | C-type lectin domain family 11 member A | 4752.854 |
| P35282\|RAB21 | 24.09 | 1 | 1 | Ras-related protein Rab-21 | 0 |
| P01786\|HVM17 | 12.96 | 1 | 1 | Ig heavy chain V region MOPC 47A | 0 |
| G3XA59\|LRC32 | 72.37 | 1 | 1 | Transforming growth factor beta activator LRRC32 | 1509.111 |
| P51885\|LUM | 38.24 | 1 | 1 | Lumican | 10629.23 |
| P17742\|PPIA | 17.96 | 1 | 1 | Peptidyl-prolyl cis-trans isomerase A | 92429.88 |
| O89020\|AFAM | 69.33 | 1 | 1 | Afamin | 118584.1 |
| Q8BH35\|CO8B | 66.19 | 1 | 1 | Complement component C8 beta chain | 0 |
| P26262\|KLKB1 | 71.34 | 1 | 1 | Plasma kallikrein | 1227.563 |
| Q7TMB8\|CYFP1 | 145.15 | 1 | 1 | Cytoplasmic FMR1-interacting protein 1 | 0 |
| O08677\|KNG1 | 73.06 | 1 | 1 | Kininogen-1 | 0 |
| P11588\|MUP1 | 20.64 | 1 | 1 | Major urinary protein 1 | 125649.9 |
| Q61171\|PRDX2 | 21.77 | 1 | 1 | Peroxiredoxin-2 | 249000.1 |
| Q61703\|ITIH2 | 105.86 | 1 | 1 | Inter-alpha-trypsin inhibitor heavy chain H2 | 1762.676 |
| P11688\|ITA5 | 114.97 | 1 | 1 | Integrin alpha-5 | 389.5701 |
| Q08857\|CD36 | 52.66 | 1 | 1 | Platelet glycoprotein 4 | 0 |
| Q8VED5\|K2C79 | 57.52 | 1 | 1 | Keratin, type II cytoskeletal 79 | 5056.65 |
| Q8BGZ7\|K2C75 | 59.70 | 1 | 1 | Keratin, type II cytoskeletal 75 | 869.7507 |
| P21956\|MFGM | 51.21 | 1 | 1 | Lactadherin | 6430.112 |
| P97350\|PKP1 | 80.84 | 1 | 1 | Plakophilin-1 | 536.9176 |
| Q07456\|AMBP | 39.00 | 1 | 1 | Protein AMBP | 27099.36 |
| P01867\|IGG2B | 44.23 | 1 | 1 | Ig gamma-2B chain C region | 80652.03 |
| Q5GFD5\|HS3S6 | 37.39 | 1 | 1 | Heparan sulfate glucosamine 3-O-sulfotransferase 6 | 1217.294 |
| Q6IRU2\|TPM4 | 28.45 | 1 | 1 | Tropomyosin alpha-4 chain | 13558.41 |
| Q9WUM5\|SUCA | 36.13 | 1 | 1 | Succinate--CoA ligase [ADP/GDP-forming] subunit alpha, m | 6847.331 |
| tr\|A0A068YAI7\|A0 | 11.68 | 1 | 1 | Expressed conserved protein | 0 |
| O09044\|SNP23 | 23.25 | 1 | 1 | Synaptosomal-associated protein 23 | 0 |
| P01942\|HBA | 15.08 | 1 | 1 | Hemoglobin subunit alpha | 88246.26 |
| P00687\|AMY1 | 57.61 | 1 | 1 | Alpha-amylase 1 | 2041.007 |
| Q61739\|ITA6 | 122.08 | 1 | 1 | Integrin alpha-6 | 14559.71 |
| Q9Z2U1\|PSA5 | 26.39 | 1 | 1 | Proteasome subunit alpha type-5 | 9768.644 |
| P00342\|LDHC | 35.89 | 1 | 1 | L-lactate dehydrogenase C chain | 25156.9 |
| Q61702\|ITIH1 | 101.00 | 1 | 1 | Inter-alpha-trypsin inhibitor heavy chain H1 | 3264.697 |
| Q61704\|ITIH3 | 99.30 | 1 | 1 | Inter-alpha-trypsin inhibitor heavy chain H3 | 7152.951 |
| P49182\|HEP2 | 54.46 | 1 | 1 | Heparin cofactor 2 | 3933.321 |
| P26041\|MOES | 67.72 | 1 | 1 | Moesin | 0 |
| P25911\|LYN | 58.77 | 1 | 1 | Tyrosine-protein kinase Lyn | 0 |
| Q64726\|ZA2G | 35.31 | 1 | 1 | Zinc-alpha-2-glycoprotein | 19803.31 |
| Q8VCS0\|PGRP2 | 57.67 | 1 | 1 | N-acetylmuramoyl-L-alanine amidase | 9157.552 |
| Q8VHL0\|UT1 | 42.10 | 1 | 1 | Urea transporter 1 | 6321.76 |
| Q8R121\|ZPI | 51.76 | 1 | 1 | Protein Z-dependent protease inhibitor | 5393.094 |
| P01637\|KV5A5 | 14.38 | 1 | 1 | Ig kappa chain V-V region T1 | 302973.4 |
| P59999\|ARPC4 | 19.65 | 1 | 1 | Actin-related protein 2/3 complex subunit 4 | 4315.371 |
| P47754\|CAZA2 | 32.95 | 1 | 1 | F-actin-capping protein subunit alpha-2 | 4033.922 |
