# Supplementary material for: Proteomic profiling of serum extracellular vesicles identifies diagnostic markers for echinococcosis
Source: PLoS Negl Trop Dis. 2022 Oct 7;16(10):e0010814. doi: 10.1371/journal.pntd.0010814 (PMC9581430; doi:10.1371/journal.pntd.0010814)
Supplement: S3 Table — (DOCX) [file pntd.0010814.s004.docx]

| Gene | Forward primer (5’ - 3’) | Reverse primer (5’ - 3’) |
| --- | --- | --- |
| VCP | GAGCTCATGTCTGCCTCGTCAGGAAATC | GTCGACCTCAACCGATGTCGTCGTAGCCAAC |
| VCP-N | GAGCTCATGTCTGCCTCGTCAGGAAATC | GTCGACCTCACGAATACAAGTCCTCATCC |

S3 Table The performance of TPx-1 and TER ATPase in diagnosis of animal echinococcosis

|  | **No. of samples** | **No. of positive samples** | | |
| --- | --- | --- | --- | --- |
|  |  | **Crude antigens** | **TPx-1** | **TER ATPase** |
| **5-day post infection** | | | | |
| Healthy mice | 64 | 0 | 3 | 1 |
| Infected mice | 64 | 0 | 4 | 4 |
| AUC (95% CI) |  | 0.617 (0.520 - 0.714) | 0.755 (0.669 - 0.840) | 0.789 (0.710 - 0.869) |
| Sensitivity (95% CI) |  | 1.56% (3.96 - 8.40) | 10.94% (4.51- 21.25) | 9.38% (3.52 - 19.30) |
| Specificity (95% CI) |  | 100.00 %(94.40 - 100.00) | 95.31% (86.91- 99.02) | 96.88% (89.16 - 99.62) |
|  | |  | P = 0.0295^a^ | P = 0.0029^a^ |
|  |  |  | P = 0.4355^b^ | |
| **6-day post infection** | | | | |
| Healthy mice | 64 | 0 | 5 | 3 |
| Infected mice | 64 | 2 | 10 | 10 |
| AUC (95% CI) |  | 0.706 (0.673 - 0.848) | 0.669 (0.569 - 0.770) | 0.744 (0.656 - 0.832) |
| Sensitivity (95% CI) |  | 1.79% (0.05 -9.55) | 12.86% (8.91- 30.40) | 20.59% (11.74 - 32.12) |
| Specificity (95% CI) |  | 100.00% (93.63 - 100.00) | 89.29% (78.12 - 95.97) | 92.86% (82.71 - 98.02) |
|  | |  | P = 0.1621^a^ | P = 0.3558^a^ |
|  |  |  | P = 0.3514^b^ | |
| **7-day post infection** | | | | |
| Healthy mice | 64 | 0 | 5 | 2 |
| Infected mice | 64 | 5 | 5 | 0 |
| AUC (95% CI) |  | 0.759 (0.677- 0.841） | 0.567 (0.467 - 0.667) | 0.5725 (0.473 - 0.672) |
| Sensitivity (95% CI) |  | 31.25% (20.24- 44.06) | 9.375% (3.52 - 19.30) | 4.688% (0.98 - 13.09) |
| Specificity (95% CI) |  | 100.00% (94.40 -100.00) | 90.63% (80.70 - 96.48) | 96.88% (89.16 - 99.62) |
|  | |  | P = 0.0024^a^ | P = 0.3113^a^ |
|  |  |  | P = 0.4132^b^ | |
| **8-day post infection** | | | | |
| Healthy mice | 64 | 0 | 2 | 1 |
| Infected mice | 64 | 0 | 7 | 12 |
| AUC (95% CI) |  | 0.6948 (0.595 - 0.795) | 0.663 (0.564 - 0.763) | 0.916 (0.861- 0.971) |
| Sensitivity (95% CI) |  | 1.79% (0.05 -9.55) | 14.29% (6.375 - 26.22) | 30.36% (18.78 - 44.10) |
| Specificity (95% CI) |  | 100.00% (93.63- 100.00) | 94.64%% (85.13 - 98.88) | 96.43% (87.69 - 99.56) |
|  | |  | P = 0.0259^a^ | P = 0.1373^a^ |
|  |  |  | P = 0.1732^b^ | |
| **9-day post infection** | | | | |
| Healthy mice | 64 | 0 | 3 | 1 |
| Infected mice | 64 | 0 | 5 | 3 |
| AUC (95% CI) |  | 0.641 (0.539 - 0.744) | 0.641 (0.539 - 0.744) | 0.767 (0.678 - 0.856) |
| Sensitivity (95% CI) |  | 1.79% (0.05 - 9.55) | 14.29% (6.38 - 26.22) | 5.357% (1.12 - 14.87) |
| Specificity (95% CI) |  | 100.00% (93.63 - 100.00) | 92.86% (82.71- 98.02) | 94.64% (85.13 - 98.88) |
|  | |  | P = 0.7350^a^ | P = 0.9848^a^ |
|  |  |  | P = 0.5930^b^ | |

Note: CI, confidence interval; ‘^a^’, statistically significant difference was compared between crude antigens and TPx-1 or TER ATPase; ‘^b^’, statistically significant difference was compared between TPx-1 and TER ATPase.
