# Supplementary material for: Proteomic profiling of serum extracellular vesicles identifies diagnostic markers for echinococcosis
Source: PLoS Negl Trop Dis. 2022 Oct 7;16(10):e0010814. doi: 10.1371/journal.pntd.0010814 (PMC9581430; doi:10.1371/journal.pntd.0010814)
Supplement: Attachment — Submitted filename: Responses to Reviewers Questions.docx [file pntd.0010814.s005.docx]

Responses to Reviewers' Questions

*Reviewer #1: Subtitle: Human serum samples:
At the beginning of M&M section, authors state ethical aspects regarding animal experiments but they did not specify anything regarding human samples.* *There any public/state/country regulation regarding this point? Do the patients have agreed and/or signed any kind of consent to the use of their sera? Have the samples be anonymized? Please add this information in “Ethic statement” section.*

Response: Thanks for your constructive comments. The following contents have been added into the subsection “Ethic statement”:

“Human studies were approved by the Research and Ethics Committee of Qinghai University Medical College (QHDX-2016–03). Written informed consent was obtained from all participants (informed consent from all adults aged 18 years and above, and parental consent for all children under 18 years old and child assent from all children aged between 7 and 17 years).”

*Subtitle: Parasite infection
Reading the entire article I realized (or I supposed) that the authors pooled the sera of 10 mice of each group giving rise to three of EV-infected mice samples and three of EV-uninfected mice samples, for each time point. Is, in fact, the experiment designed in this way?*

Response: Yes, it is right.

*In the same sense, in line 105 it is stated that the sera of 10 uninfected mice were harvested together. But as the sentence only refers to uninfected mice, I did not understand the reason of pooling just those samples. It is possible that in this sentence the EV-infected mice were involuntary omitted? Please re-write this section to clarify from the very beginning which groups are going to be compared and if the experiment is well-designed.*

Response: Thanks for your constructive comments. The subsection has been re-written: “The experimental design was based on two settings and mice were intraperitoneally injected with *E. multilocularis* protoscoleces as described previously [18]. A ﬁrst experimental infection of mice with *E. multilocularis* was established for EV isolation. Six to eight-week-old male BALB/c mice were divided randomly into two groups. One group (n= 60) was intraperitoneally injected with 1,000 E. multilocularis protoscoleces and the other group (n= 60) was inoculated with phosphate-buffered saline (PBS) solution as a control. The infected mice were euthanized at 30- (n=20), 60- (n=20), and 90-day (n=20) post infection and whole blood samples were collected for serum isolation, respectively. The uninfected mice at each sampling time point (n=20) were used as controls. The sera of 10 mice of each group were pooled, giving rise to three of EV-infected mice samples and three of EV-uninfected mice samples for each time point. A second experimental infection of mice with E. multilocularis was designed for assessment of the enzyme-linked immunosorbent assay (ELISA) established in the study. Male BALB/c mice (n= 64) were injected with E. multilocularis protoscoleces as above and 50 μL of blood were collected by tail vein bleeding at 0-, 5-, 6-, 7-, 8-, 9-,10-, 15-, 20-, and 30-day post infection and added into the microcentrigue tubes containing 50 μL physiological saline. The pre-infection sera (at 0-day post infection) were used as uninfected controls. Samples were centrifuged at 2,000×g for 10 min at 4 °C. Serum samples were immediately transferred into a clean tube and frozen at −80 °C for late use.”

*Subtitle: Purification and characterization of EVs
The authors describe the purification of EV. Finally the EV are resuspended either in PBS or denaturation buffer. I wonder if the authors perform any additional step to lyse the EV and to recover protein samples to be used in proteomic studies. I think that this step is mandatory. Only resuspending in any denaturating buffer can lead to membrane lyses but much better yield when a specific lysis procedure is performed.*

Response: Thanks for your constructive comments. We agree with you and indeed used extra steps to lyse the EVs. For example, sonication and boiling steps were included for preparation of EVs samples for Western blotting.

S*ubtitle: Protein digestion and mass spectrometry
The authors describe reduction and alkylation reaction routinely carried in MS sample prep. Normally 10 mM DTT is used and an excess of IAA (typically 50-55 mM). The reason for IAA excess is to assure that react with all sulfhydryl group in proteins and in the reaction mixture. Remember that DTT has two sulfhydryl groups and for that reason at least the concentration of IAA normally used is at least twice. Please confirm if the concentration of IAA expressed in the text is correct.*

Response: Thanks for your comments. We confirmed that the concentration of IAM was 55 mM.

*In the digestion step:
1- Specify the brand and quality of trypsin used and the relation enzyme to proteins used.*

Response: Yes, they have been specified.

*2- The authors add a subsequent additional step of digestion. Trypsin used for proteomic studies is very robust and efficient enzyme. This is not the common procedure. Do the authors have any particular evidence of incomplete protein digestion that explain this extra step?*

Response: Thanks for your comments. We agree with you. In fact, we only conducted trypsin digestion.

*For nanoHPLC specify the following parameters:
• How is the relation of ACN/FA in mobile phase A and B.*

Response: Mobile phase A was water containing 0.1% formic acid/2% acetonitrile. The mobile phase B was acetonitrile containing 0.1% FA.

*• Which column was used in nano-HPLC system (diameter, length, size of pore, C18).*

Response: The desalted peptides were subjected to nanoLC-MS/MS on tandem self-packed C18 column (20 cm in length×7.5 μm internal diameter, pore size of 3 μm).

*• Gradient used in nano-LC system.*

Response: As suggested, we have added: “An increasing linear gradient of mobile phase B was used as follows: 0-5min, 5 % phase B; 5–90 min, 5–25% phase B; 90–100 min, 25–35% phase B; 100–110 min, 35%- 80% phase B; 115–116 min, 80–5% phase B, 116-120min, 5% phase B”.

*For MS analysis specify the following parameter.
MS voltage setting, capillary temperature.*

Response: As suggested, we have added: “For data acquisition, the mass spectrometer parameters were set as follows: the spray voltage of 2.3 kV and spray interface temperature of 160 °C”.

*Full mass scan parameters: ranges of m/z acquisition, resolution.*

Response: The m/z range was 350~1,250 m/z with resolving power of 600,000. This information has been added.

*Fragmentation parameters: number TopN fragmented ions in each duty cycle, collision energy, and resolution.*

Response: The dynamic elimination of the parent ion was set below: within half of the peak time (about 12 seconds), the fragmentation of the same parent ion did not exceed 2 times. The collision energy was set as 'Rolling Collision Energy”.

*Subtitle: Protein identification
Add the following information:
• The dates when de protein databases were downloaded.
• Fixed and variable modification of peptides.*

Response: For protein identification, protein databases were downloaded from Uniprot ([Release 2022/02](https://www.uniprot.org/help/downloads)). Carbamidomethylation of cysteine (Cys) was used as a fixed modification, and methionine oxidation was used as a variable modification.

*Subtitle: Preparation of recombinant proteins
Does any tag was added to protein sequence in the cloning process to assure protein purification? If yes, please clarify this point.*

Response: Yes, a His-tag is fused to the N-terminus of the target proteins.

*Subtitle: statistical analysis
It is clear in other section of M&M and later in the paper, but it should be more clarifying if you detail which are the “three groups” mentioned in this section (30 d, 60 d, and 90 d infected and uninfected mice?).*

Response: Yes, they are. The information has been added.

*Reviewer #2: Please state the source and concentration of trypsin used for the digests.*

Response: The information has been added.

*Line 226 the term “exosomal” here is incorrect. We do not know what type of vesicles have been isolated here -in all likelihood there will be a mix of vesicle types pelleted by ultracentrifugation. I think “EV” should be used instead (here and elsewhere in the manuscript).*

Response: We agree with you and have corrected it.

*Figure 2 – immunogold labelling. No methods for this are described – please add.*
Response: The information has been added.

*Reviewer #3: The manuscript by Guo and colleagues describes serum-derived extracellular vesicles screened in model-organisms and patients infected with Echinococcus multilocularis, aiming in an early detection of the infection. The study design is appropriate. However, a few details are not described in sufficient details to follow all the conclusions of the manuscript.*

*Line 107ff. Please provide more details about the human samples. How are the parasite cysts staged? Which IHA test was performed. Which follow-up marker was used and how were the patients verified to be “cured”? For example an Em18-test should be performed, as this is currently the best marker for viable and non-proliferative infections (see the corresponding PLoS NTD article: https://doi.org/10.1371/journal.pntd.0010146, which should be included in the discussion). Furthermore, it has been also shown, that various tests (like the Em18), but also other tests are not good for the early and sub-clinical detection of the parasite (see* [*https://doi.org/10.3390/pathogens11050518*](https://doi.org/10.3390/pathogens11050518)*).*

Response: Thanks for your constructive comments. The currently standardized clinical classification of hydatids [cysts](http://www.baidu.com/link?url=OYJuBzveUUZZsfz66qFPovAmkEprKJDa2Ldxl2xFrcpTI0yEIuToJ7ypbZvPMGiNKTXyoXt-fQ53NQF9NC7bpa) is the [classification scheme](https://www.sciencedirect.com/topics/engineering/classification-scheme) reached by consensus in the World Health Organization Working Group of Experts on Hydatidosis (WHO/IWGE) based on Gharbi ultrasound typing. All cases had cyst (hydatid lesion of > 5 cm in diameter) in the liver. Positive test for indirect red blood cell [agglutination test](https://www.sciencedirect.com/topics/medicine-and-dentistry/agglutination-test) (Indirect [haemagglutination test](https://www.sciencedirect.com/topics/medicine-and-dentistry/hemagglutination-test), IHA) was performed. The cured patients were confirmed by ultrasound. As suggested, we have added the results using Em18-based ELISA.

*Line 114ff. According to the MISEV guidelines on how studies on extracellular vesicles should be described (https://doi.org/10.1080/20013078.2018.1535750), ultracentrifugation only provides an enrichment of the particles. Therefore, this should be clearly stated, that this is an “enrichment” and not a purification. As this level of purity is sufficient for the further conclusions, some hits (like TGF-b) are probably still serum-derived and not from the host EV cargo. This should definitively be discussed according to the guidelines.*

Response: Thank for your valuable suggestion. We have revised the subsection title as “Enrichment and characterization of EVs” and exosome as “EV”. We have discussed TGF-β: “Extensive proteomics of exosomes has verified the presence of TGF-β1 in EVs [29,30], which was recently localized on the surface of mast cell-derived EVs [31].”

*Line 142ff. Please provide the genome and UniProt release number*

Response: We have added the related information.

*Line 165. Provide the number of used rabbits.*

Response: We have added the related information.

*Line 179. For what purpose where X-ray films used? Please describe the imaging, as ECL is usually a chemiluminescent dye.*

Response: T[hermo Scientific Pierce ECL Plus Substrate](https://www.thermofisher.cn/order/catalog/product/32132) is an acridan-based chemiluminescent and chemifluorescent HRP substrate for Western blotting using X-ray film or CCD- or laser-based imagers. So the sentence has been changed into “Subsequently, the membranes were washed and incubated with goat anti-rabbit IgG secondary antibody (1: 10,000, KPL) followed and were visualized by exposing X-ray ﬁlms using Pierce ECL Western Blotting Substrate (ThermoFisher Scientific)”.

*Line 180ff. Please describe the ELISA in more details. Which sera were used? From which species in which experiment? How many samples per group? How were they collected?*

Response: To evaluate the diagnostic values of animal echinococcosis using two antigens, sera were obtained from 64 mice at 0-, 5-, 6-, 7-, 8-, 9-, 10-, 15-, 20-, and 30-day post infection, respectively. The pre-infection sera (at 0-day post infection) were used as control. Mouse bloods (50 μL) were collected by tail vein bleeding and added into 1.5 mL microcentrifuge tubes containing 50 μL of physiological saline. Samples were centrifuged at 2,000×g for 10 min at 4 °C.

To evaluate the diagnostic values of human AE using two antigens, a total of 94 sera of echinococcosis patients and 59 cured patients were collected. A total of 65 serum samples from healthy volunteers were used as negative control. Whole blood samples were collected. For serum isolation, the blood samples were collected and placed at room temperature for 2 h. Samples were centrifuged at 2,000×g for 10 min at 4 °C. Serum samples were immediately transferred into clean tubes and frozen at −80 °C.

All the information was detailed in the subsections “Parasite infection and sample collection” and “Human serum samples”.

*Line 194ff. Please describe how the positive/negative threshold was determined? Was it by a ROC and maximal youden’s index or by S/N values?*
Response: For each antigen, the positive cut-off values were calculated by the OD value in each test serum (S) divided by the mean OD value of control sera (N). Samples with an S/N value more than 2 were considered to be positive.

*Results
Reviewer #1: Subtitle: Mouse protein components in serum-derived EVs
Page 10, line 219: Since all proteomic analysis software apply statistical analysis is more correct to express “were statistically differentially abundant…” or “show a statistically difference in relative abundance…”.*

Response: We have revised.

*Subtitle: Parasite-origin protein components in serum-derived EVs
In this section the authors describe a series of proteins that are consistently identified in EV obtained from infected mice. They do not correspond with the most abundant statistically differential proteins identified in the first result’s section. The proteins describe do not necessarily have to be the most abundant, but… they are identified as statistically differential? Please, add a comment referring to this point.*

Response: Thank for your valuable suggestion. Statistically, it is impossible to determine whether parasite-origin proteins are differential because they are not detected in the uninfected samples.

*Most importantly, the author designed a mice experiment accounting considerable number of infected and uninfected mice and performed a comparative proteomic analysis. As a result, they described the presence of protein detected in E. multilocularis infected-EV mice sera in all time points studied. But they do not mention at all if those proteins are detected or not in uninfected samples. The rest of the paper centers in the validation of those proteins that are described as good proteins for diagnosis and prognosis. For the reasons mentioned above I consider really important to know if the selected proteins are detected in the uninfected samples. Please describe, as detailed as possible, this point in this result section (include this information in the main text and tables). the selected proteins are detected in the uninfected samples*

Response: Thank for your constructive suggestions. The parasite-origin proteins were exclusively detected in the infected samples. We have added this point in the result section (Table 2).

*It should be very interesting also that the validation experiments by WB include EV derived from uninfected mice and confirm the absence of signal. Consider that uninfected samples are the best negative control to the conclusion of the present work*

Response: The pre-infection sera (at 0-day post infection) were used as uninfected control. Western blotting analysis showed that TPx-1 and TER ATPase were absence in EVs isolated from pre-infection sera.

*The authors test the immunogenicity of those proteins using sera of infected mice. In this section it would also be absolutely interesting to study this response in uninfected mice samples as the best negative control. Experiment using uninfected mice sera should be performed, present in the main text, figures and discussion section.*

Response: Thank for your valuable suggestion. The pre-infection sera (at 0-day post infection) were used as uninfected control.

Figure legends:
*Fig.1: Figure captions should understandable by itself without the need to come back to main text. Specify that CD63 is an exosomal marker. Also, when “those proteins” is written, is better to name it in the legend text.*

Response: Thank for your valuable suggestion. We have revised.

*Reviewer #2: Figure 2. I assume antibody-conjugated gold probes were used as secondary antibody and that this is what the arrow is pointing to in Figure 2? The legend should be revised to include more description. What is the difference between Fig 2A and Fig 2B? Do they both show the same thing? I can only see a single probe (arrowed) which is not very convincing. Additional images showing more probes, specifically clustered on the EVs, is required.*

Response: Thanks for your constructive comments. That is right. Gold-labeled secondary antibodies (Jackson Immunoresearch, dilution 1:20) were used. The red arrows indicated the parasite-derived EVs. Immune electron microscopy of serum-derived EVs isolated from *E. multilocularis*-infected mice (Fig.2A, right) and uninfected mice (Fig.2A, left) were performed using anti-Em14-3-3 antibodies. The image showing more probes clustered on the parasite-derived EVs were reloaded.

*A mouse 14-3-3 protein was also identified in the EVs (Table S2). How do you know if the Em-14-3-3 antibody is specific for parasite-derived EVs? Some images where this antibody has been applied to serum EVs from uninfected mice should be included.*

Response: Thank for your valuable suggestion. Our previous study showed that Em-14-3-3 antibody was specifically recognized with native Em-14-3-3(15). Western blotting result showed that the Em-14-3-3 signal was only detected in the serum-derived EVs isolated from the *E. multilocularis*-infected mice while the signal was not detected in the serum-derived EVs isolated from the uninfected mice (Fig. 2D). Coincidently, the immunoelectron microscopy result showed that Em-14-3-3-labed EVs was only detected in the serum-derived EVs isolated from the *E. multilocularis*-infected mice (Fig 2A).

15. Zheng Y, Guo X, Su M, Guo A, Ding J, et al. (2017) Regulatory effects of *Echinococcus multilocularis* extracellular vesicles on RAW264.7 macrophages. Veterinary parasitology 235: 29-36.

*Figure 2 legend – panel D is not described.*

Response: We have added.

*Have the authors tried immunolocalization using their antibodies on tissue sections of E. mulitlocularis in situ within host tissues? It might help to understand how the parasite is releasing EVs and how they end up in the host blood.*

Response: Thank for your valuable suggestion. [Immunofluorescence](http://www.baidu.com/link?url=fwuQdXmc47APTExw0Y2u0LoE-RE0MRNd-K5-NlrcFDOQFIKNhd_inMMeYgU1sVKnJ0sn__O-1SqFjPYdDJhWRMGSjsIOsd7norfvuwO9pYbuvHV3RFXfNyTSwGMkiVxa) localization showed that both TER ATPase and TPx-1 proteins were localized in the liver areas close to parasitic lesions (Fig. 2C).

*Reviewer #3: Line 214. Please specify, that CD63 was an mammalian EV-marker, as there is several evidence, that the tetraspanins are not cross-reacting between helminth and other species. See e.g. Sotillo et al., 2020:* [*https://doi.org/10.1016/j.ijpara.2020.04.010*](https://doi.org/10.1016/j.ijpara.2020.04.010)

Response: We have revised.

*Line 274. The crude Em antigen is known not to be a valid marker for follow-up. Rather use the state-of art diagnostics for distinguishing viable vs non-productive infections, such as the global available Em18 antigen. This must be included to draw the conclusions to define “cured” patients.*

Response: Thank for your valuable suggestion. As suggested, we have added the results of Em18-based ELISA.

*Conclusions
Reviewer #1: Conclusions are partially supported by the data presented. Very relevant conclusion are obtained. The authors engage in a big work, using a beautiful set of samples (EV from sera of a total of 120 mice –infected and uninfected-, and sera from sick and healthy humans), but negative samples obtained from uninfected mice is not discussed and is not used as negative control in very relevant experiments. In my opinion, those samples are the best negative control and the authors do not take advantage of it. The authors should repeat the experiments using those extremely valuable samples.
Also, the proteomic detection of the protein selected as relevant for prognosis and diagnosis in negative samples must be discussed in Results and Discussion sections.*

Response: Thank for your valuable suggestions. In this study, the pre-infection mouse sera (at 0-day post infection) were used as control. The uninfected samples were underlined in the result and discussion sections.

*Reviewer #2: Generally OK but more could be added if the suggested proteomics comparisons were included.*

Response: Thank for your valuable suggestion. We have added as suggested.

*Reviewer #3: The data presented by Guo and colleagues provide important results to diagnose accurately this important disease in humans. Some limitations are described. Some aspects however are missing and should be included:
It is somehow unclear, why the important EV protein “14-3-3” was not considered as a candidate, beeing a known and specific EV-marker. Is there any further data on this? Please comment on that, as this protein is stated in the abstract as one of the promising candidates, but on which no further experiments were performed.*

Response: Our previous study showed low specificity of 14-3-3 in the diagnosis of experimental murine AE.

*Line 288 / 295. There is no data showing the purity of EV’s. The used technique only allow to enrich EVs. A contamination with soluble excreted/secreted proteins is probable, such as for example the abundance of TGFb. Further details about purity and working with helminth EVs are provided in Sotillo et al., 2020: https://doi.org/10.1016/j.ijpara.2020.04.010, and the MISEV2018 guidelines (*[*https://doi.org/10.1080/20013078.2018.1535750*](https://doi.org/10.1080/20013078.2018.1535750)*).*

Response: Thank for your valuable suggestion. We have revised the subsection title as “Enrichment and characterization of EVs”. Moreover, we have discussed TGF-β1: “Extensive proteomics have verified the presence of TGF-β1 in EVs [29,30], which was recently localized on the surface of mast cell-derived EVs [31].”.

*Line 321. The early diagnosis was only shown in murine samples, which were infected i.p., which is a highly artificial model. Therefore, this conclusion should be put into perspective of the used system.*

Response: Thank for your valuable suggestion. We have revised.

*Reviewer #1:*

*Main page; Abstract: Echinococcus in italics.
E. multilocularis in italics
E. multilocularis –infected mice, without space between multilocularis and mid-dash.*

Response: We have revised.

*Page 1, line3: Wang with capital letter.*

Response: We have revised.

*Page 5, line 88: “proteomic” instead of “proteomics”.*

Response: We have revised.

*Page 5, line 96: “animal” instead of “Animals”.*

Response: We have revised.

*Page 6, line: 116: “group” instead of “groupe”.*

Response: We have revised.

*Page 6, line: 124: “1:5” instead of “1: 5”, without space.*

Response: We have revised.

*Page 6, line 131: is better to used IAM as an abbreviation of iodoacetamide, since IAA is frequently used also to iodoacetic acid. To avoid confusion, please use IAM.*

Response: We have revised.

*Page 7, line 132: express the temperature as 37 °C, with an space between the number and the unit.*

Response: We have revised.

*Page 7, line 151: add a space between “<” and “0.05”.*

Response: We have revised.

*Page 8, line 172: add a space between “4” and “°C”, and delete the space between “1” and “:” in “1:10,000”.*

Response: We have revised.

*Page 8, line 173: delete the space between “USA)” and “,”.*

Response: We have revised.

*Page 9, line 183, 185, 186: Add a space between the number and “°C”.*

Response: We have revised.

*Page 9, line 186, 188: delete the spaces between “:” and the number that follows.*

Response: We have revised.

*Page 22, line 462: “from” instead of “fromr”.*

Response: We have revised.

*Page 28, line 533: “E. multilocularis” in italics.*

Response: We have revised.

*Reviewer #2: Some areas might need to be checked for grammar/spelling.*

Response: We have double-checked the MS thoroughly.

*Reviewer #3: Line 70: E. multilocularis and E. granulosus sensu latu are the only causative agents, and not the most important ones. Please change this.*

Response: We have revised.

*Line 81. It is right, that EVs are first suspected to be a garbage-disposal system, however, there is plenty of evidence, that they are specific interactors. I suggest to change the sentence to “EVs were orginally identified in reticulocytes as a supposed garbage-disposal mechanism.*

Response: As suggested, we have revised.

*Line 95. Please change the sentence to “has been approved by the ...”*

Response: We have revised.

*Line 116. An “r” is missing in “group”*

Response: We have added.

*Line 130. Please write “EV proteins” (EV without “s”)*

Response: We have revised.

*Line 154. Please delete the “o”*

Response: We have deleted.

*Line 167. The IgG are polyclonal. Therefore, not only specific antibodies are purified by Prot G*

*Sepharose. Therefore, “specific” should be deleted.*

Response: We have deleted.

*Line 259. I suggest adding the p-value, and omit in the table 3-*

Response: We have added.

*Line 266. I suggest deleting “approximately”, as it was exact the stated number.*

Response: We have deleted.

*Line 267. I would suggest adding the p-value (p=0.002), and omit in the table*

Response: We have added.

*Line 271. It is rather a “prognostic value” as a “diagnostic value”*

Response: We have revised.

*Line 325. Just write “is suitable for early diagnosis of human echinococcosis”.*

Response: We have revised.

*Figure 2. Please indicate in the figure capture, what the red arrow means. The approach of immune-gold labelling is missing in the material and methods sections and should be described in more details.*

Response: We have added.

*Summary and General Comments
Use this section to provide overall comments, discuss strengths/weaknesses of the study, novelty, significance, general execution and scholarship. You may also include additional comments for the author, including concerns about dual publication, research ethics, or publication ethics. If requesting major revision, please articulate the new experiments that are needed.
Reviewer #1: The paper describes the identification, by a proteomic approach, of two relevant proteins useful for early diagnosis and prognosis of echinococcisis. The data is validated by different methodologies. The experimental design is very well performed using EV derived from E. multilocularis infected and uninfected mice. They use a significant number of animals and different time points. They also use human sera from sick and healthy humans. In principle, it seems a wonderful work, but unfortunately the authors do not uses the uninfected mice proteomic data in discussion. Also they do not take advantage of these invaluable samples as the best negative control for all the validation strategies. I strongly consider that negative samples derived resulted must be discussed and also consider that those sample should be used in validation experiments as negative controls.*

Response: Thank for your valuable suggestion. The uninfected samples were underlined in result and discussion sections.

*Reviewer #2: Given the amount of proteomic data that is available for EV from related species (E. granulosus), either purified EVs or isolated from mouse sera, cyst fluid etc (see links to examples below – there are others) the authors have missed an opportunity for comparison of parasite-derived EVs detected in host fluids.* [*https://pubmed.ncbi.nlm.nih.gov/35360110/*](https://pubmed.ncbi.nlm.nih.gov/35360110/)

Response: Thank for your valuable suggestion. We have added.

*Also, it would be useful to compare the proteome of EVs directly recovered from E. multilocularis following in vitro culture (https://pubmed.ncbi.nlm.nih.gov/28215864/) with those isolated from serum of infected mice. A significant overlap would provide more support that parasite-derived EVs are actually present in the mouse serum described here.*

Response: Thank for your valuable suggestion. We have added.

*Reviewer #3: The manuscript is well presented, and the topic and work are definitively interesting for the journals’s readers and suitable to be published in PLoS NTD with some modifications. An early detection of alveolar echinococcosis is missing and would improve the treatment management. The data are presented clearly, and the manuscript is well written.
Human ethic approval is missing and should be amended, including a statement, that the consent of the patients is present.*

Response: Thank for your valuable suggestion. We have added.
