# Supplementary material for: Proteomic profiling of serum extracellular vesicles identifies diagnostic markers for echinococcosis
Source: PLoS Negl Trop Dis. 2022 Oct 7;16(10):e0010814. doi: 10.1371/journal.pntd.0010814 (PMC9581430; doi:10.1371/journal.pntd.0010814)
Supplement: Attachment — Submitted filename: Responses to Reviewers Questions.docx [file pntd.0010814.s006.docx]

Responses to Reviewers' Questions

Reviewer #1: I would like to thank the authors for addressing all of my concerns in the M&M section. I found the experimental design and methodology much more clear.

However, I still have some minor comments.

Line 122: As both set of mice (infected and unifected) were used for further experiments, I find better to delete "the infected mice" and write only "Mice were euthanized..."

Response: We revised.

Line 150: In the previous version of the manuscript it was explained that EVs-containing pellets were resuspended either in PBS or denaturing solution. In the present text the denaturing solution was omitted. I wonder why since it is a better solution to extract proteins from vescicles. And I still do not know if a protein extraction procedure was employed for proteomic analysis. Please, clarify this point.

Response: For western blotting, EVs-containing pellets were indirectly lysed in RIPA buffer and then quantified using the Bradford assay, subsequently added 4×SDS-PAGE loading buffer”.

For proteomic analysis, EVs-containing pellets were extracted using RIPA buffer with protease inhibitor Cocktail, and then quantified using the Bradford Assay.

Line 171: If protein extraction procedure was carried out it could also be described here.

Response: We have added. For proteomic analysis, EVs-containing pellets were extracted using RIPA buffer with protease inhibitor Cocktail, and then quantified using the Bradford Assay.

Line 171: I think that it is tidier to write the sentence "Protein concentration was measured...", before reduction and alkylation procedure description.

Response: Yes, it is right. EVs-containing pellets were extracted using RIPA buffer with protease inhibitor Cocktail, and then quantified using the Bradford Assay.

Line 173: It is described that 100 ug of proteins were digested (this is a quite big quantity of proteins for protomic analysis).

Response: Usually, 100 μg of proteins were digested. However, the quantity of EV proteins from each group was not up to100 μg. In this study, 10 μg of EV proteins from each group were digested. We have revised.

Please specify in line 176, how much protein was injected into the mass spectrometer.

Line 179: Since the gradient described here is not only the increase phase B gradient for protein separation, but also the gradient to let the column in a standby state (a phase B decreasing gradient), please delete the word "increase".

Response: We deleted.

Line 258: I asked for the specification of the "three groups" here. In the answer to the reviewers it is stated that it was done. But I didn't find it in this section. Please add it.

Response: we revised.

Data availability: the deposited data is not fully available.

Response: This dataset should be publicly released. We have contacted the original repository yet in order to trigger the public release.

Reviewer #2: (No Response)

Reviewer #3:

Line 117. First sentence still unclear, what is the intension. Maybe better to write (if my suggestion is correct) "Mice were injected at two different time points intraperitoneally with E. multilocularis protoscoleces as described previously"

Response: Thank for your valuable suggestion. We have revised.

Line 124ff. If sera of 10 mice of each group were pooled, this gives duplicates (and not triplicates) for each timepoint. Please clarify.

Response: No triplicates in each time point.

Line 191ff. Please profide the identifier of the mice database and which genome was used for E. multilocularis. I guess it is PRJEB122? I suggest following sentence "mouse protein database (88,027 sequences) obtained from Uniprot (Release 2022/02), and E. multilocularis genome and protein databases PRJEB122 (10,971 sequences; retrieved from WormBase ParaSite)".

Response: Thank for your valuable suggestion. We have revised.

Line 220. Please describe, what kind of tissue sections. Which tissue, origin, ...

Response: We have revised.

Results

Reviewer #1:

Line 287: Please, also explain the behavior of AMY1 in the WB analysis.

Response: Thank for your valuable suggestion. The expression of AMY1 was examined by WB analysis (Fig 1F). We added the described in revised MS.

Line 297 or 306: Explain the reason(s) why the protein 14-3-3 was excluded from further analyses.

Response: 14-3-3 protein has been selected for validation by Western blotting (Line 297, Fig. 2B). TPx-1 and TER ATPase were selected for further analyses, so 14-3-3 was no done the [immunofluorescence](http://www.baidu.com/link?url=fwuQdXmc47APTExw0Y2u0LoE-RE0MRNd-K5-NlrcFDOQFIKNhd_inMMeYgU1sVKnJ0sn__O-1SqFjPYdDJhWRMGSjsIOsd7norfvuwO9pYbuvHV3RFXfNyTSwGMkiVxa) localization analyses.

Line 317: Is is possible that "fig. 2" here is in fact "fig. 3"?

Response: We have revised.

Regarding my request to add a statistics for the proteins uniquely detected in infected samples, I checked MQ software and I must recognized that I couldn't find the option to do that. I just want to comment that the software I use to proteomic analysis (doi: 10.1038/s41596-022-00690-x) has this option. I think that it is a good option. I know that biologically, is not possible to found parasite proteins in unifected samples. But it is technically possible. First of all, you can find false positive (this is the reason why we apply a FDR rate value for the acceptance of proteins, and the authors did it). But also, sometimes it is recommended to mix control and samples of interest, because in long runs columns show drift in its performance. In this kind of run set up, this bias is considered. However, some abundant proteins of one group (either using appropiate blanks) can be detected in the other set of samples, usually with in one replicate or with vert low signal or spectral counts. The software I describe take this issue into account, and can statistically determine that a protein is present just in one set of sample.

Response: Thank for your valuable suggestion.

Reviewer #2: (No Response)

Reviewer #3: No further comments

Reviewer #1: Considering changes made along the manuscript, I find this section very well written. Every aspect of the results is discussed and conclusions are very well understood.

Reviewer #2: (No Response)

Reviewer #3: No further comments

Reviewer #1: Line 50: delete the space between ") ,".

Response: We have revised.

Line 77: "identify of diagnostic", delete "of".

Response: We have revised.

Line 140: Add an space between ELISA and [.

Response: We have revised.

Line 160: "Briefly" insted of "Brefily".

Response: We have revised.

Lines 184-185 and everywhere is written: m/z in italics.

Response: We have revised.

Line 185: add a space between the number and "m/z".

Response: We have revised.

Line 222: To be consistent in the way of PBS-T is written latter in the text, write here "TBS-T".

Response: We have revised.

Line 223: Add a space between "study)" and "After". The letter A is missing in "Alexa".

Response: We have added.

Line 228: "Protein samples" with lower case.

Response: We have revised.

Line 269: Add a space between "1A" and "and".

Response: We have revised.

Line 273: Detele the "n" from "an".

Response: We have revised.

Line 273: The word enriched is writen twice very closely in the same sentence. I suggest to replace the first "enriched" whit "more abundant".

Response: We have revised.

Line 351: Add a space between "EVs" and "[".

Response: We have revised.

Line 351: I believe that it is "most" instead of "mast".

Response: We have revised.

Line 388: Add a space between "follow-up" and "[50].

Response: We have revised.

Line 390: Delete one of the two "the".

Response: We have revised.

Line 408: Delete one of the two dots written together.

Response: We have revised.

In the text and legends the protein "G-Patch domain containing 8" is abbreviated as GPATCH8, but in figures as "GPTC8". Please unify the nomenclature.

Response: We have revised.

Reviewer #2: (No Response)

Reviewer #3: Author summary:

Line 77ff. This sentence is a bit complicated. Maybe change to "This study signify the role of EVs for the identification of iagnostic candidates by the discovery of two identified proteins, TER ATPas and TPX-1. First results indicate their diagnostic and prognostic values in experimental murine and human echinococcosis."

Materials and methods:

Line 121. A "comma" is missing before ", and the other group [...]"

Response: We have revised.

Line 129. Please write "blood samples" instead of "bloods"

Response: We have revised.

Line 130. A "comma" is missing before the "and": "post infection, and [...]"

Response: We have revised.

Line 133. "until further use" instead of "for late use"

Response: We have revised.

Line 136. "clinical" instead of "clinica"

Response: We have revised.

Line 144. "until further use" instead of "for late use"

Response: We have revised.

Line 160. "Briefly, [...]"

Response: We have revised.

Results:

Line 273. Here, the word "enriched" is not needed.

Response: We have revised.

Discussion:

Line 348. "EV marker"

Response: We have revised.

Line 357. remove "including blood". It is not really needed.

Response: We have revised.

Summary and General Comments

Reviewer #1: With the changes made the manuscript has improved a lot. All sections are much better understood, leading to a better understanding of the conclusions.

Reviewer #3: This is a second review of the manuscript submitted by Guo and colleagues. Early diagnosis of echinococcosis is missing. Therefore, the demonstrated work will provide valuable information which will guide for future point of care diagnostics to control this devastating infection disease. The revised manuscript include now more information to follow the methods and conclusions.
